# Correction: The Gastroprotective Effect of *Vitex pubescens* Leaf Extract against Ethanol-Provoked Gastric Mucosal Damage in Sprague-Dawley Rats

**DOI:** 10.1371/journal.pone.0179072

**Published:** 2017-05-31

**Authors:** Nahla Saeed AL-Wajeeh, Mohammed Farouq Halabi, Maryam Hajrezaie, Summaya M. Dhiyaaldeen, Daleya Abdulaziz Bardi, Suzy M. Salama, Elham Rouhollahi, Hamed Karimian, Rojin Abdolmalaki, Ainnul Hamidah Syahadah Azizan, Hapipah Mohd Ali, Suzita Mohd Noor, Mahmood Ameen Abdulla

The affiliation for the first author is incomplete. Nahla Saeed AL-Wajeeh is affiliated with the following: Department of Biomedical Science, Faculty of Medicine, University of Malaya, Kuala Lumpur, Malaysia; Department of Molecular Medicine, AL-Gumhouri Teaching Hospital, Ministry of Public Health & Population, Sana'a, Yemen; Ministry of Higher Education & Scientific Research, Yemen.

The following information is missing from the funding section: This study was supported grants from the University of Malaya (PG054-2012, RP043A/15HTM and RP021D/14AFR).
